# Characteristics of mass-forming autoimmune pancreatitis commonly misdiagnosed as a malignant tumor

**DOI:** 10.3389/fsurg.2023.1017621

**Published:** 2023-01-25

**Authors:** Si Chen, Hanlei Zhang, Fang Fang, Chao Ye, Kaiguang Zhang

**Affiliations:** ^1^ Department of Gastroenterology, the First Affiliated Hospital of USTC, Division of Life Sciences and Medicine, University of Science and Technology of China, Hefei, China; ^2^ Department of Gastroenterology, the Affiliated Provincial Hospital, Anhui Medical University, Hefei, China

**Keywords:** autoimmune pancreatitis, immunoglobulin g4, carbohydrate antigen 19-9, endoscopic ultrasonography, differential diagnosis

## Abstract

**Objective:**

This study aimed to explore the clinical characteristics and differential diagnosis of patients with autoimmune pancreatitis (AIP) and pancreatic cancer to prevent misdiagnosis and mistreatment.

**Methods:**

The clinical data of patients with AIP with suspected pancreatic or bile duct malignancy and pancreatic cancer were retrospectively analyzed. The risk factors and the diagnostic value of IgG4 and Tbil levels before treatment for AIP was investigated. Moreover, the imaging features and response to hormone therapy were analyzed.

**Results:**

AIP was commonly observed in men. Compared to patients with pancreatic cancer, the incidence of poor appetite and weight loss and carbohydrate antigen 19-9 (CA19-9) level was lower in patients with AIP, while the immunoglobulin G4 (IgG4) level was higher (*p *< 0.05). After treatment, the IgG4 and CA19-9 levels in patients with AIP were decreased (*p *< 0.001). IgG4 level before treatment (OR = 2.452, 95%CI: 1.180–5.096, *P* = 0.016) and total bilirubin (Tbil) level before treatment (OR = 0.992, 95%CI: 0.985–0.998, *P* = 0.013) were independent risk factors of AIP. Furthermore, the diagnostic value of IgG4 level before treatment, Tbil level before treatment, IgG4/Tbil before treatment, and a combination of these indicators was high. Moreover, 15 (68.18%) patients with AIP had space-occupying lesions of the pancreas, and 16 (72.73%) had autoimmune cholangitis. Most patients with AIP were sensitive to hormone therapy.

**Conclusions:**

The Tbil and IgG4 levels, imaging findings, and hormone therapy reactivity could differentiate AIP from pancreatic cancer. A combination of IgG4, Tbil, and IgG4/Tbil before treatment might be a promising diagnostic biomarker for AIP.

## Introduction

In 1961, Sarles reported a case of chronic inflammatory sclerosing pancreatitis with hyperglobulinemia (1). In the next 30 years, similar cases were reported occasionally worldwide. In 1995, Yoshida proposed the concept of autoimmune pancreatitis (AIP) to describe this type of chronic pancreatitis associated with autoimmune symptoms (2). AIP is a rare and special type of chronic pancreatitis mediated by autoimmunity, and its main characteristics include mild-to-moderate abdominal pain, obstructive jaundice (progressive or intermittent), enlarged pancreas, irregular pancreatic duct stenosis, and lymphocyte infiltration (3). AIP is often accompanied by diabetes or other autoimmune diseases.

AIP can be classified into two subsets (AIP-I and AIP-II) based on clinical and histological characteristics. AIP-I, also known as immunoglobulin G4-related diseases (IgG4-RD), is characterized by the presence of several IgG4-positive lymphoplasmic cells infiltrating the affected pancreatic and extra-pancreatic tissues and interstitial striate fibrosis. AIP-I patients often present with IgG4-related sclerosing cholangitis, autoimmune sialadenitis, autoimmune nephritis, and retroperitoneal fibrosis, particularly obstructive jaundice caused by cholangitis (4). AIP-II is a type of idiopathic ductal pancreatitis, with granulocyte epithelial disease accompanied by pancreatic duct destruction and occlusion, and it is not correlated to IgG4. AIP-I accounts for most AIP cases in Asian patients (5). Due to the low incidence and changing epidemiological characteristics of AIP, knowledge about this condition is still limited; clinical guidelines for diagnosis and treatment are mostly based on consensus among experts (6). Clinical practice significantly varies per country, particularly in Asia and North America/Europe.

There are two major problems in the current diagnosis and treatment strategies for AIP. First, the clinical manifestations of AIP are not specific, and laboratory indicators, imaging characteristics, and clinical prognosis are extremely similar to those of malignant tumors in the pancreas and bile duct. The incidence of pancreatic/bile duct cancer is significantly higher than that of AIP (7). Moreover, there have been some reports of the misdiagnosis of AIP as pancreatic cancer (8). The patients undergo operative treatment and pancreatic resection, which may result in increased surgical trauma and postoperative complications and waste more medical resources compared to medical treatment. An experience in China has revealed that AIP accounts for 49% of the chronic pancreatitis population undergoing surgical treatment, confirming that AIP patients are always subjected to unnecessary surgical resection owing to its similarity in clinical features with pancreatic cancer (9). Conversely, the misdiagnosis of malignant tumors as AIP would delay the best opportunity for treatment. Second, clinicians often perform excessive auxiliary examinations due to a lack of experience and confidence in managing rare diseases. Even in patients with typical clinical manifestations and serum indicators, endoscopic retrograde cholangiopancreatography (ERCP) or endoscopic ultrasonography are still performed repeatedly. Therefore, a better understanding of the characteristics of AIP is helpful to guide the diagnosis and treatment of AIP.

Comparing Chinese and Western patients, obstructive jaundice was the most frequent initial symptom (68% vs. 43%), and the elevation of serum IgG4 was more significant (10). In this retrospective study, we assessed the clinical manifestations such as jaundice, laboratory indicators such as serum IgG4, imaging findings, pathological features, and treatments of patients with AIP who were highly suspected of having pancreatic/bile duct malignant tumors upon admission. Our study aimed to identify a simple and reliable diagnostic method that applied to the Chinese population.

## Materials and methods

### Patients

Between January 2016 and December 2019, 22 patients with AIP and 30 patients with malignant pancreatic tumors treated at our hospital were enrolled in this retrospective study. In total, 21 patients with AIP underwent imaging examinations, including computed tomography (CT) scan, magnetic resonance imaging (MRI), and endoscopic ultrasound (EUS), and only 11 patients underwent EUS-guided fine needle aspiration (EUS-FNA) due to factors such as medical costs, patient compliance, and puncture risk. Patients with AIP met the International Consensus Diagnostic Criteria (11), and those with malignant tumors, connective tissue diseases, and other subtypes of pancreatitis with similar clinical manifestations, such as typical idiopathic pancreatitis or painful chronic pancreatitis, were excluded. Patients with pancreatic cancer were diagnosed based on postoperative pathological examination or tissue puncture pathology findings and classified according to the American Joint Committee on Cancer (AJCC) TNM staging system**.** The patients selected in this study were all preoperatively staged less than IIA and without lymph node or distant metastasis or other pancreatic tumors. The reason for the selection of patients with pancreatic cancer as the control group was that, compared to other pancreatic diseases, patients with pancreatic cancer had a higher relative mortality rate and a worse prognosis, and misdiagnosis of pancreatic cancer may result in poor clinical outcomes. This study was approved by the Ethics Committee of the First Affiliated Hospital of the University of Science and Technology of China (USTC) (No. 2020-RE-004).

### Data collection

Baseline data (demographic characteristics, age, first symptoms, and organ involvement), radiological findings (CT scan/MRI/magnetic resonance cholangiopancreatography/ERCP), histopathological characteristics, serum indicators (albumin [ALB], hemoglobin [HB], alanine aminotransferase [ALT], aspartate aminotransferase [AST], gamma-glutamyl transferase [GGT], alkaline phosphatase [ALP], total bilirubin [Tbil], IgG4, and carbohydrate antigen 19-9 [CA19-9] levels), and response to hormone therapy among patients with pancreatic cancer were analyzed and compared in detail.

### Statistical analysis

Quantitative data with normal distribution are expressed as mean ± standard deviation (SD) and analyzed with *t*-test, whereas quantitative data with abnormal distribution are presented as median (P25, P75) and were analyzed using the Mann–Whitney U test. Enumeration data are presented according to composition ratio and were analyzed using the chi-square test. To analyze the risk factors of AIP, clinical factors, including sex, age, clinical symptoms (abdominal discomfort, jaundice (the Tbil concentration is more than 17.1 μmol/L), weight loss (defined as the weight loss of at least 3 kg or 8% of basal body weight within 1 month) and poor appetite), CA19-9 level before treatment, IgG4 level before treatment, and Tbil level before treatment, were included in the univariate and multivariate logistic regression analysis. Odds ratio (OR) and 95% confidence interval (CI) were used to calculate the effect size. The diagnostic value of IgG4 level before treatment, Tbil level before treatment, IgG4/Tbil before treatment, a combination of IgG4 and Tbil before treatment, and a combination of IgG4, Tbil, and IgG4/Tbil before treatment was analyzed using receiver operating characteristics (ROC) analysis. All statistical data were processed using SPSS 17.0 (SPSS Inc, Chicago, Illinois, USA). A *P*-value < 0.05 was considered statistically significant.

## Results

### Baseline data of the participants

Among patients with AIP, there were 20 men and 2 women, with a sex ratio of 10:1. The average age was 61.82 ± 13.59 years. Moreover, 20 cases were clinically diagnosed as AIP-I type, and 2 cases as AIP-II type. Due to various factors, such as medical cost, individual compliance, and concern on the risk of aspiration biopsy, only 11 patients underwent EUS-FNA, and 21 patients underwent imaging examinations, including abdominal ultrasound, CT, and MRI. Among patients with pancreatic cancer, 20 were men and 10 were women, with a sex ratio of 2:1, and the average age was 62.83 ± 7.42 years. There was no difference in terms of age between the two groups (*P* = 0.996). However, AIP was commonly observed in men (*P* = 0.032).

The initial symptoms of the two groups were abdominal discomfort (bloating and abdominal pain), jaundice, poor appetite, and weight loss. The incidence rates of abdominal discomfort, jaundice, poor appetite, and weight loss were 54.54% (*n* = 12), 45.45% (*n* = 10), 13.64% (*n* = 3), and 27.27% (*n* = 6) in patients with AIP, and 73.33% (*n* = 22), 36.67% (*n* = 11), 63.33% (*n* = 19), and 53.33% (*n* = 16) in patients with pancreatic cancer, respectively. There was no difference in terms of abdominal discomfort (*P* = 0.236) and jaundice (*P* = 0.576) between the two groups. However, the risk of poor appetite was significantly lower in patients with AIP than that in patients with pancreatic cancer (13.64% vs. 63.33%, *P* = 0.001). The incidence rate of weight loss was significantly lower (27.27% vs. 53.33%, *P* = 0.049) and the degree of weight loss (4.5 ± 3.9 kg vs. 8.3 ± 5.7 kg, *P* = 0.028) was lower in the AIP group than in the pancreatic cancer group (Table 1).

**Table 1:** Demographic characteristics and symptoms of AIP patients and pancreatic cancer patients.

	AIP patients (*n* = 22)	Pancreatic cancer patients (*n* = 30)	*p* value
Demographic characteristics and symptoms			
Male/female	20/2	20/10	0.032
Age (years)	61.82 ± 13.59	62.83 ± 7.42	0.996
BMI	21.71 ± 2.46	19.77 ± 7.11	0.263
HB	121.50 ± 17.14	118.59 ± 19.49	0.574
ALB	37.29 ± 5.16	39.14 ± 2.65	0.110
ALT	113.0 (35.8, 163.5)	48.0 (20.4, 164.5)	0.28
AST	74.5 (45.3, 118.8)	40.5 (22.0, 130.8)	0.21
GGT	167.2 (46.0, 473.3)	372.0 (64.3, 880.1)	0.027
ALP	135.5 (80.1, 449.5)	309.5 (162.0, 557.5)	0.046
Tbil	153.0 (37.4, 215.8)	185.2 (52.9, 473.4)	0.038
Poor appetite	3	19	0.001
Jaundice	10	11	0.576
Abdominal discomfort	12	22	0.236
Weight loss	6	16	0.049
Weight loss (kg)	4.5 ± 3.9	8.3 ± 5.7	0.028

BMI, Body Mass Index; ALB, albumin; HB, haemoglobin; ALT, alanine aminotransferase, AST, aspartate aminotransferase, GGT, gamma-glutamyl transferase; ALP, alkaline phosphatase; Tbil, total bilirubin.

### Serological indicators

The average serum IgG4 level in patients with AIP was 8.9 (3.9, 12.7) g/L, and it was elevated in 19 (86.36%) cases. The increase in IgG4 levels in 17 (77.27%) patients with AIP was more than twice the normal upper limit. In patients with pancreatic cancer, the average serum IgG4 level was 0.60 (0.41, 1.18) g/L, with a slight increase observed in only two cases (3.05 and 2.38 g/L). The serum IgG4 level of patients with AIP was significantly higher than that of patients with pancreatic cancer (*P* < 0.001). After treatment, the serum IgG4 level in patients with AIP decreased significantly (*P* = 0.001).

In total, 7 of 22 patients with AIP were positive for CA19-9, accounting for 31.82% of all cases. Meanwhile, 27 of 30 patients with pancreatic cancer were positive for CA19-9, with a positivity rate of 90.00%. The mean CA19-9 values of patients with AIP and pancreatic cancer before treatment were 32.5 (16.5, 118.6) and 231.0 (90.8, 1126.5) U/mL, respectively. This result showed that the CA19-9 level before treatment in patients with AIP was significantly lower than that in patients with pancreatic cancer (*P* = 0.011). The CA19-9 levels in patients with AIP decreased significantly after treatment (*P *< 0.001), while there was no significant difference in CA19-9 levels before and after treatment in patients with pancreatic cancer (*P* > 0.05).

There was a significant difference in terms of GGT, ALP, and Tbil levels between the two groups (*P *< 0.01) but no significant difference in ALB, HB, ALT, and AST levels (*P* > 0.05) (Table 1).

### Risk factor analysis

Univariate and multivariate regression analyses were conducted to analyze the risk factors of AIP (Table 2). The results showed that sex, age, clinical symptoms, and CA19-9 before treatment had no statistical significance on the risk of AIP. IgG4 level before treatment (univariate regression analysis: OR = 2.059, 95%CI: 1.041-4.073, *P* = 0.038; multivariate regression analysis: OR = 2.452, 95%CI: 1.180–5.096, *P* = 0.016) and Tbil before treatment (univariate regression analysis: OR = 0.961, 95%CI: 0.928–0.9961, *P* = 0.028; multivariate regression analysis: OR = 0.992, 95%CI: 0.985–0.998, *P* = 0.013) were the independent risk factors for AIP.

**Table 2:** Univariate and multivariate regression analyses of the risk factors of AIP.

Variables	B	S.E.	Wald	df	Sig.	Exp (B)	95% CI
Univariate regression analysis							
Sex	4.760	3.032	2.464	1	0.117	116.699	0.306–44488.792
Age	-0.130	0.104	1.549	1	0.213	0.878	0.716–1.077
Poor appetite	-1.300	1.813	0.514	1	0.473	0.273	0.008–9.516
Weight loss	-0.279	1.973	0.020	1	0.888	0.757	0.016–36.191
Icterus	-0.682	1.732	0.155	1	0.694	0.506	0.017–15.072
Abdominal discomfort	1.068	2.009	0.283	1	0.595	2.910	0.057–149.364
CA19-9 before treatment	-0.003	0.002	1.849	1	0.174	0.997	0.993–1.001
IgG4 before treatment	0.722	0.348	4.308	1	0.038	2.059	1.041–4.073
Tbil before treatment	-0.039	0.018	4.801	1	0.028	0.961	0.928–0.996
Constant	9.332	6.694	1.944	1	0.163	11,291.778	
Multivariate regression analysis							
IgG4 before treatment	0.897	0.373	5.779	1	0.016	2.452	1.180–5.096
Tbil before treatment	-0.008	0.003	6.207	1	0.013	0.992	0.985–0.998
Constant	-1.431	1.368	1.094	1	0.296	0.239	

AIP, autoimmune pancreatitis; IgG4, immunoglobulin G4; CA19-9, carbohydrate antigen 19-9; Tbil, total bilirubin; 95% CI, 95% confidence interval.

### The diagnostic value of IgG4 and Tbil levels before treatment for AIP

**Figure 1:**
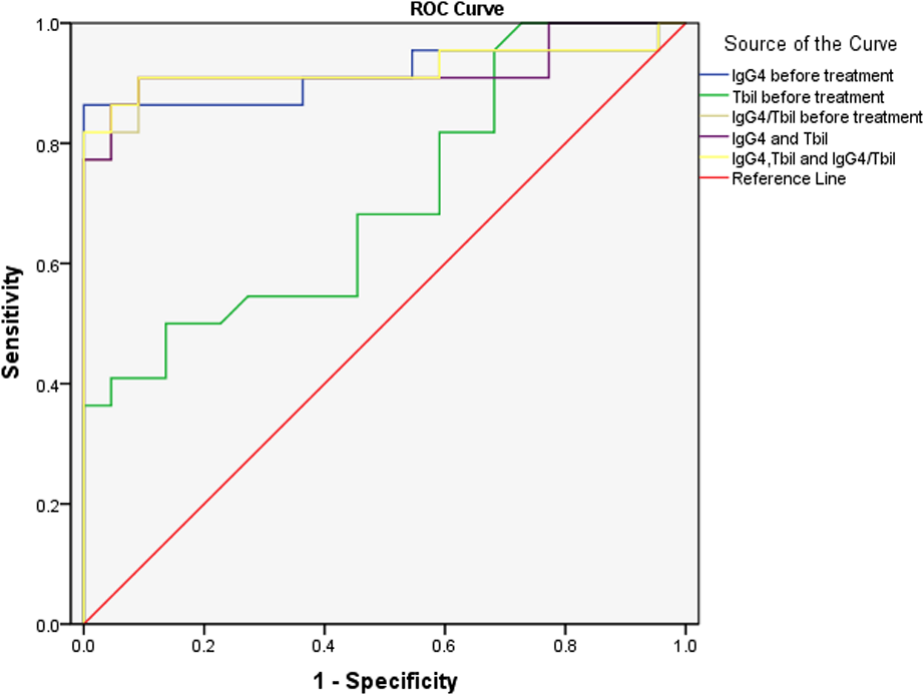
ROC analysis of IgG4 level before treatment and tbil level before treatment in the diagnosis of AIP. ROC, receiver operating characteristics; IgG4, immunoglobulin G4; Tbil, total bilirubin; AIP, autoimmune pancreatitis.

Further ROC analysis showed the area under the ROC curve (AUC) of IgG4 level before treatment, Tbil level before treatment, IgG4/Tbil before treatment, a combination of IgG4 and Tbil before treatment, and combination of IgG4, Tbil, and IgG4/Tbil before treatment was 0.915, 0.707, 0.919, 0.921, and 0.924, respectively (Figure 1, Table 3), indicating that all of these indicators had a high diagnostic value for AIP, among which the combination of IgG4, Tbil, and IgG4/Tbil before treatment had the highest diagnostic value.

**Table 3:** ROC analysis of IgG4 level before treatment and Tbil level before treatment in the diagnosis of AIP.

Variables	Area	Std. Error	Sig.	95% CI
IgG4 before treatment	0.915	0.051	0.000	0.816–1.000
Tbil before treatment	0.707	0.078	0.019	0.553–0.860
IgG4/Tbil before treatment	0.919	0.050	0.000	0.822–-1.000
IgG4 and Tbil	0.921	0.049	0.000	0.826–1.000
IgG4, Tbil and IgG4/Tbil	0.924	0.050	0.000	0.826–1.000

ROC, receiver operating characteristics; IgG4, immunoglobulin G4; Tbil, total bilirubin; 95%CI, 95% confidence interval.

### Imaging features

CT scan, MRI, and EUS revealed that 15 (68.18%) patients with AIP had a focal pancreatic mass. Of them, 13 (86.67%) had a mass in the pancreatic head or the uncinate process, and 2 (13.33%) in the pancreatic body or tail. The remaining 7 (31.82%) patients presented with an enlarged or diffusely enlarged pancreas. Sixteen (72.73%) patients with AIP had a mass in the pancreatic head and diffused inflammation accompanied by common bile duct narrowing, upper intrahepatic bile duct dilatation, and bile duct wall thickening. EUS revealed the typical bilateral sign of the common bile duct in some patients. Seven (31.82%) patients with a focal pancreatic mass, particularly those with a pancreatic head lesion, were highly suspected of having a malignant tumor at diagnosis. However, experienced physicians can clearly diagnose diffuse AIP *via* radiological examination, and misdiagnosis seldom occurs (Table 4).

**Table 4:** Radiologic features of AIP patients and pancreatic cancer patients.

Computed tomography scan/magnetic resonance cholangiopancreatography/endoscopic ultrasound	AIP patients (*n* = 22)	Pancreatic cancer patients (*n* = 30)	*p* value
Swelling	8	1	0.001
Space-occupying lesion	15	30	0.004
Changes in the pancreatic duct	12	14	0.229
Bile duct wall thickening	16	5	0.001
Bile duct stenosis of the pancreatic segment and choledochectasia above the pancreatic level	16	19	0.075
Diagnoses of other diseases	7	1	0.003

AIP, autoimmune pancreatitis.

**Figure 2:**
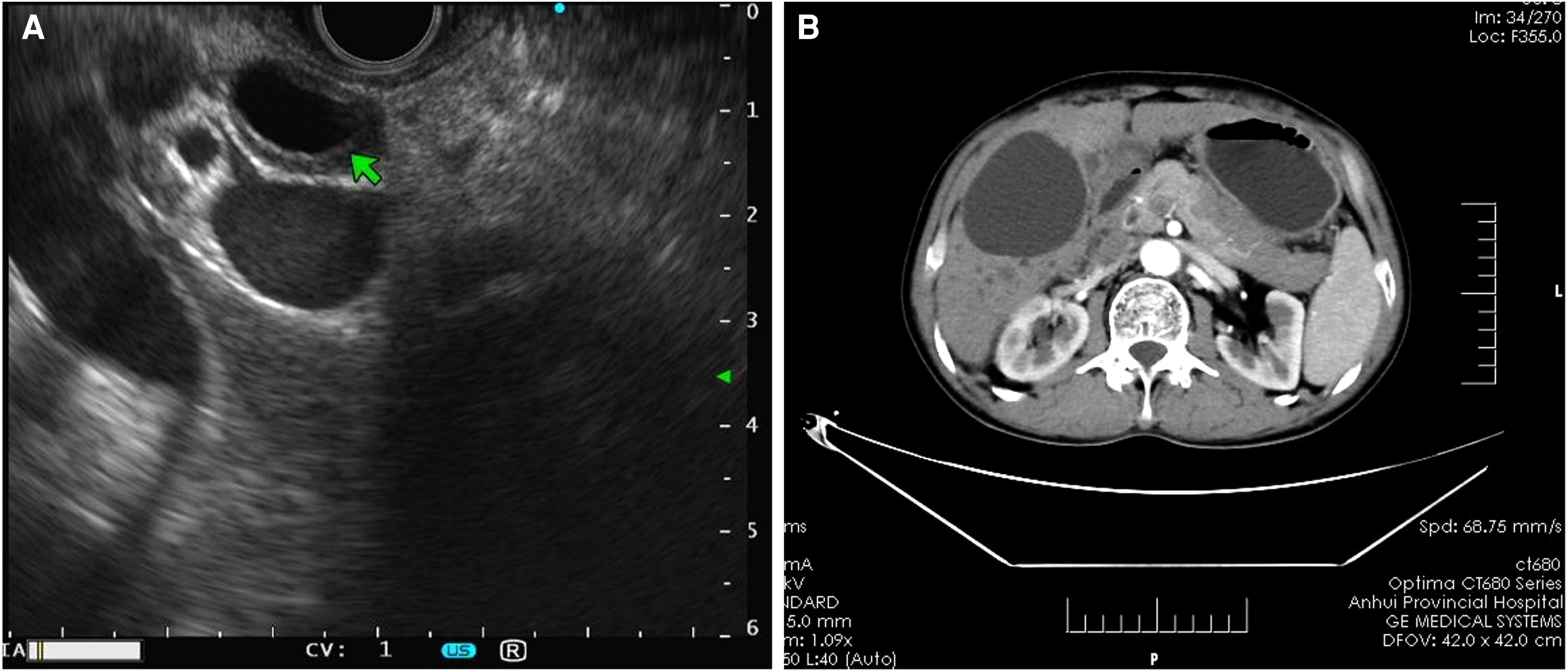
Imaging features of AIP. (**A**) Endoscopic ultrasound revealed bile duct wall thickening and common bile duct dilatation with the bilateral sign in patients with AIP. (**B**) Computed tomography scan showed an even thickening of the common bile duct wall, evident enhancement in the arterial phase, and uneven and irregular enhancement of the head and neck areas. AIP, autoimmune pancreatitis.

Patients with AIP and pancreatic cancer experienced lymph node hyperplasia and swelling in the abdominal cavity and retroperitoneum (*P* > 0.05). In 16 (72.73%) patients with AIP, lesions were detected in the bile ducts, which were accompanied by autoimmune cholangitis. Only 3 (10.00%) patients with pancreatic cancer had lesions in the common bile duct. There were significant differences in bile duct wall thickness and tube occupation between patients with AIP and patients with pancreatic cancer. The typical EUS images of AIP showed bile duct wall thickening, pancreatic mass lesions, irregular narrowing of the main pancreatic duct, and bile duct dilatation (Figure 2).

Among patients with AIP, four presented with elevated blood glucose, one with posterior peritoneal occupancy, one with ulcerative colitis, and one with systemic lupus erythematosus (female). Although the AIP-I type often involves multiple extra-pancreatic glands, including lacrimal and salivary glands, no extra-pancreatic glands were involved in the patients in this study, which might be due to the small sample size and limited detection methods. None of the abovementioned lesions were found in patients with a tumor.

### Response to hormone therapy

Patients with AIP who received hormonal therapy at a starting dose of 30 mg/day for 2 weeks achieved good response. After treatment, clinical symptoms, including abdominal discomfort, jaundice, poor appetite, and weight loss, improved significantly in 15 patients. The serum IgG4 and Tbil levels decreased from 8.9 (3.9, 12.7) mg/L and 153.0 (37.4, 215.8) mmol/l before treatment to 2.0 (0.8, 3.7) mg/L and 31.0 (17.1, 58.5) mmol/L after treatment, respectively (*P* < 0.001) (Table 5).

**Table 5:** Serological values in AIP before and after treatment.

	Before treatment	After treatment	*p* value
IgG4 level	8.9 (3.9, 12.7)	2.0 (0.8, 3.7)	0.001
Tbil level	153.0 (37.4, 215.8)	31.0 (17.1, 58.5)	< .001

AIP, autoimmune pancreatitis; IgG4, immunoglobulin G4; Tbil, total bilirubin.

Four other patients with AIP who were not eligible for hormone therapy received conservative treatment, including liver protection and anti-inflammatory therapy. Surveillance revealed that their symptoms and level of serum indicators also improved to some extent.

### Follow-up

During the later period of follow-up, three patients with AIP presented with jaundice and fluctuations in IgG4 levels. Patients with contraindications were excluded, and azathioprine was administered at an initial dose of 50 mg/day for normal-weight patients and 100 mg/day for heavy-weight patients. Patients with pancreatic cancer received chemotherapy or underwent surgery. Jaundice and abdominal discomfort improved within 1 year of follow-up after surgery. However, no significant improvement was noted in patients who received chemotherapy.

## Discussion

IgG4-RD is a chronic, progressive inflammatory disease with fibrosis, and it can involve multiple organs such as the pancreas, kidneys, skin, lungs, and peritoneum. AIP-I is a special type of IgG4-RD, and it accounts for most AIP cases, with an incidence rate of 70%–90% (12). The misdiagnosis of AIP as pancreatic ductal adenocarcinoma will cause physical and psychological trauma to patients. Moreover, the misdiagnosis of atypical pancreatic cancer with AIP characteristics will lead to treatment delay and extremely poor outcomes. Therefore, this study summarized the clinical characteristics and differential diagnosis of patients with AIP and pancreatic cancer to prevent misdiagnosis and mistreatment.

Wu et al. analyzed the clinical manifestations of patients with AIP. They reported that the first typical symptoms of patients with AIP were mainly mild-moderate upper abdominal pain and jaundice (13). A few patients would develop new diabetes (14), insufficient pancreatic exocrine (15), or acute pancreatitis (16). Especially in AIP-I patients, jaundice was more common due to IgG4-associated sclerosing cholangitis (17, 18).

Abdominal pain and distension, jaundice, poor appetite, and weight loss were the main initial characteristics of patients with AIP in this study, and these findings were consistent with those of other reports (19, 20). However, jaundice and abdominal pain are not AIP-specific symptoms and are common in pancreatic cancer. Jaundice fluctuated and sometimes improved spontaneously in patients with AIP (21). However, jaundice caused by pancreatic duct stenosis in pancreatic cancer often shows progressive exacerbation, indicating that fluctuating jaundice might be an indicator to distinguish AIP from pancreatic cancer (22, 23).This study found significant differences in the incidence of jaundice between patients with AIP and patients with pancreatic cancer and fluctuation of bilirubin levels in patients with AIP during the different courses of the disease. Naitoh et al. analyzed the clinical manifestations of patients with AIP-I and pancreatic cancer, and the results showed that there was no difference in the incidence of jaundice between AIP-I patients and pancreatic cancer patients, which was not consistent with our findings (24). This may be related to the differences in sampling patients and the characteristics between the East and the West. In the study of Naitoh et al., the incidence of abdominal pain was significantly higher in patients with pancreatic cancer than that in patients with AIP, and our findings do not support this result. The incidence of poor appetite and weight loss was lower in patients with AIP than those in patients with pancreatic cancer.

AIP-I has a predominance among Asians, and its diagnosis is based on typical imaging findings and serum IgG levels (25). Meanwhile, in Europe and America, the two types of AIP are common, and the diagnosis focuses more on histology (26). The clinical manifestations of AIP are diverse but not specific and are extremely similar to those of pancreatic cancer, particularly in those with space-occupying pancreatic lesion and obstructive jaundice, which makes the differential diagnosis extremely difficult (27, 28). In this study, 7 (32.82%) patients with space-occupying lesions in the pancreas were highly suspected of having a malignant tumor during the early stage of diagnosis. However, in a previous study, more than half of AIP-I patients had external pancreatic involvement, and inflammatory bowel disease was observed in a few patients with AIP-I and AIP-II (29), particularly AIP-II patients with ulcerative colitis (30). All these findings provide valuable information for clinicians regarding the diagnosis of AIP. Approximately 72.73% of patients with AIP had lesions in the extra-pancreatic bile duct, while these lesions were observed only in 10.00% of patients with pancreatic cancer (31).

IgG4 plays an important role in the diagnosis of AIP. A meta-analysis revealed that IgG4 had a high accuracy in differentiating AIP from chronic pancreatitis and pancreatic cancer (32). Some studies have shown that the positivity rate of IgG4 in patients with AIP in China is up to 86.0% (33). A small number of patients with pancreatic cancer have elevated IgG4 level (34). Thus, the diagnosis of pancreatic cancer should not be completely excluded. However, the serum IgG4 level in patients with pancreatic cancer is usually <2.8 g/L (35). Ghciale et al. have found that considering a threshold of 2.8 g/L, the sensitivity and specificity of IgG4 in diagnosing AIP were 53% and 99%, respectively (36). In this study, the serum IgG4 level was elevated in approximately 86.36% of patients with AIP, and it was more than twice the normal upper limit in 77.27% of patients. Only 6.67% of patients with pancreatic cancer had a slight increase in IgG4 levels, indicating that the incidence and degree of IgG4 level increase were significantly different between the two diseases. Moreover, multivariate logistic regression analysis revealed that IgG4 level before treatment was an independent risk factor of AIP, indicating the association between IgG4 level before treatment and the risk of AIP. Some studies have shown that although IgG4 has a high specificity in diagnosing AIP, its sensitivity is insufficient. Particularly in cases of a mass-like lesion in the pancreatic head in AIP, the lesion is difficult to distinguish from locally advanced pancreatic head cancer (37). Thus, IgG4 should be used in combination with other markers. In our study, Tbil before treatment was also identified as an independent risk factor of AIP. ROC analysis showed that the AUC of IgG4 level before treatment, Tbil level before treatment, IgG4/Tbil before treatment, a combination of IgG4 and Tbil before treatment, and combination of IgG4, Tbil, and IgG4/Tbil before treatment was 0.915, 0.707, 0.919, 0.921, and 0.924, respectively (Figure 1, Table 3), indicating that all of these indicators had a high diagnostic value for AIP, among which the combination of IgG4, Tbil, and IgG4/Tbil before treatment had the highest diagnostic value.

CA19-9 is the most effective indicator for the diagnosis and follow-up of patients with pancreatic cancer (38). A CA19-9 level > 150 U/ml is highly correlated to pancreatic cancer (39). In patients with space-occupying AIP, the CA19-9 level should be considered an important indicator. Our results showed that the incidence and degree of CA19-9 elevation were significantly different between patients with AIP and patients with pancreatic cancer, and the CA19-9 level of patients with AIP significantly reduced to a near-normal level after treatment. Despite this, CA19-9 level was not an independent risk factor of AIP.

Meanwhile, the CT scan features of the tumor were low-density foci, mild enhancement in the arterial phase of scanning, a lower degree of enhancement than that of the normal pancreatic parenchyma, and progressive enhancement in the portal and delayed phases (40). The pancreatic duct lesions in patients with AIP were mainly stenosis, which could manifest as single or multiple tunneling stenoses, and the proximal pancreatic duct was not dilated or only slightly dilated (41). The pancreatic duct was longer in AIP, and the proximal pancreatic duct dilation was less in AIP than in pancreatic cancer (42).

EUS can be used to assess pancreatic lesions at a close range and even detect recurrent lesions earlier than can be detected using conventional imaging examinations (43). In AIP patients with IgG4-related sclerosing cholangitis, a high-low-high sandwich-like echo pattern could be found in the affected bile ducts, and a relatively clear boundary is often observed between AIP and normal tissues (44). Moreover, the characteristics of AIP include irregular narrowing of the pancreatic duct and wall thickening, and these results were consistent with the study of Kamisawa et al. (45). In this study, 11 patients with AIP were pathologically analyzed using different puncture methods (mainly guided by endoscopic ultrasonography and color Doppler ultrasound). However, the positive rate of pathological diagnosis was relatively low, which might be influenced by various factors such as the quality of the aspiration or puncture specimen and the professional limitations of pathologists.

Hormone therapy is the first choice for the treatment of AIP. Most guidelines recommend prednisone at a dose of 40 mg or 0.6 mg/kg to induce remission (46, 47). In patients with mild symptoms or diabetes, the dose can be reduced accordingly to 30 mg or 0.5 mg/kg (48). The clinical symptoms (including involvement of external organs of the pancreas) are considered as treatment indications. After two weeks of treatment with an initial dose of 30 mg/day among eligible patients, most clinical symptoms significantly improved. However, a small number of untreated patients also presented with some spontaneous improvement, indicating that the condition had a self-limiting characteristic. Some patients underwent ERCP before the placement of internal stent drainage (*n* = 4). However, the outcome was poor, and the jaundice did not subside.

In this study, only two patients with AIP had positivity to IgG4 based on immunohistochemistry examination. The coincidence rate of pathological results based on international diagnostic standards was only 33.3%, which was affected by the quality of specimen tissues, condition of the pathology laboratory, and subjective factors of pathologists. In this study, the international consensus was adopted as the standard, and the diagnosis of AIP was mainly based on serology, imaging, and laboratory examination findings and therapeutic effects. The patients with AIP and pancreatic cancer differed in clinical manifestations (poor appetite and weight loss), serological indicators (positivity rate, higher degree of CA19-9 and IgG4 elevation, and intrahepatic and extrahepatic cholestasis), imaging findings, and response to hormone treatment. The joint application was essential in improving the actual diagnostic rate of AIP.

However, there are some limitations to this study. First, this was a single-center clinical study. Second, this swas a retrospective study, and the representativeness of the statistical analysis was limited. Third, the small sample size and significant differences in individual data affected the reliability of our results and their clinical application. Hence, future studies should be conducted to overcome these limitations.

In summary, serum Tbil, IgG4/Tbil, and IgG4 levels before treatment, combined with specific clinical symptoms, like appetite and weight loss, typical EUS findings, and response to hormone therapy, can help differentiate AIP from pancreatic cancer. The combination of IgG4, Tbil, and IgG4/Tbil levels before treatment may be a promising diagnostic biomarker for AIP.

## Data Availability

The original contributions presented in the study are included in the article/**Supplementary Material**, further inquiries can be directed to the corresponding author/s.
